# Noise Immunity and Robustness Study of Image Recognition Using a Convolutional Neural Network

**DOI:** 10.3390/s22031241

**Published:** 2022-02-06

**Authors:** Vadim Ziyadinov, Maxim Tereshonok

**Affiliations:** Science and Research Department, Moscow Technical University of Communications and Informatics, 111024 Moscow, Russia; m.v.tereshonok@mtuci.ru

**Keywords:** neural networks, pattern recognition, noise in imaging systems, robustness, training dataset, noise immunity

## Abstract

The problem surrounding convolutional neural network robustness and noise immunity is currently of great interest. In this paper, we propose a technique that involves robustness estimation and stability improvement. We also examined the noise immunity of convolutional neural networks and estimated the influence of uncertainty in the training and testing datasets on recognition probability. For this purpose, we estimated the recognition accuracies of multiple datasets with different uncertainties; we analyzed these data and provided the dependence of recognition accuracy on the training dataset uncertainty. We hypothesized and proved the existence of an optimal (in terms of recognition accuracy) amount of uncertainty in the training data for neural networks working with undefined uncertainty data. We have shown that the determination of this optimum can be performed using statistical modeling. Adding an optimal amount of uncertainty (noise of some kind) to the training dataset can be used to improve the overall recognition quality and noise immunity of convolutional neural networks.

## 1. Introduction

Deep learning and big data analytics are, nowadays, important fields in computational science. Various organizations face the necessity of bringing these areas into their work processes to keep up with current trends. Deep learning neural networks can identify the most complex patterns in the data quickly and efficiently at high levels of abstraction, while those patterns are not observed in the first approximation. Information from machine learning applications can deepen the understanding of many hidden processes, as well as solve problems of prediction and automation in many areas of life, such as speech recognition [1], computer vision [2,3], and data visualization [4].

Automatic pattern and image recognition technologies have the widest applications in image processing. The use of convolutional neural networks (CNNs) increases its success for image processing, character and handwritten text recognition [5], license plate recognition [6], human, plant, and animal pathology detection [7,8], face and emotion recognition [9,10], distinguishing objects of interest in a video stream [11], etc.

Most publications deal with new datasets from various problem domains [7,8,12]. Many publications are concerned with neural network topology and learning technique improvements [13]. However, there are many unsolved problems in image recognition tasks. First, *the recognition accuracy is sometimes poor or insufficient*. False diagnoses made by a neural network, while not being a big problem nowadays (since the data obtained from the network are verified by the operator), can be a barrier toward expanding the impact of automatic recognition algorithms in the future. The same might be said for automatic driving systems, such as automobile autopilots.

**Figure 1:**
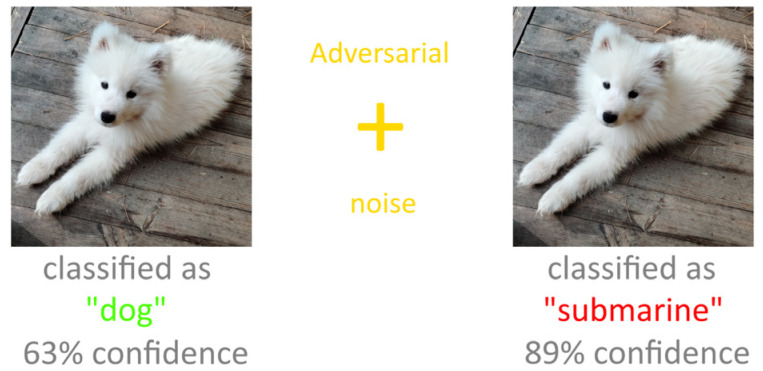
An example of an adversarial attack on a convolutional neural network.

Second, the results of neural networks are affected by data distortions, such as adversarial attacks [14,15]. Figure 1 shows an example of this kind of attack: an image of a dog with added (invisible to the human eye) noise is recognized by the network as an image of a submarine with a large coefficient of confidence. Thus, *images with different amounts of distortions are recognized with different accuracies*.

Third, *there is no universal approach to estimate the optimality and robustness* of a trained neural network. It cannot be predicted in advance how the trained neural network will behave when new data are obtained, and we cannot be unequivocally sure that the network will correctly recognize new data, especially if statistical characteristics of new data differ from those of the data used for training.

Recent publication analyses showed that robustness studies are performed only in terms of precision–recall curve plotting [16]. Some publications deal with the estimation of adversarial attack success rates rather than with increasing noise immunity of the neural networks [17]. Recent works concerning uncertainty quantifications in neural networks have not provided solutions for increasing neural network noise immunity [18]. A noise immunity study of a neural network is still “out of consideration”. However, such an investigation seems to be the key to solve adversarial attack problems and to improve the robustness and correctness of recognition of various data by neural networks.

The most important aspect in the neural network application is training, and its success mainly depends on the correct training data representation. Complex and large neural networks trained on poorly represented data, in addition to their high resource consumptions, turn out to be much poorer than simple networks that are trained on correct and well-organized data [19,20]. As a result, feature construction—the correct feature generation process (for the training data)—is the most important part of machine learning.

Correct and deep feature construction involves the potential of the CNN’s generalization ability, which can (and should) be utilized to withstand noise added to the recognized data and even adversarial attacks. Noise-immune CNN should gather the information from its layers, where the noise influence on the detectable features is quite low. For example, to fight high-frequency noise, the CNN should utilize the features obtained in its deep convolutional layers, where input data are averaged throughout large regions of the original image. We suppose that training CNNs using noisy data can help to improve their generalization abilities and capabilities of withstanding adversarial attacks.

**Figure 2:**
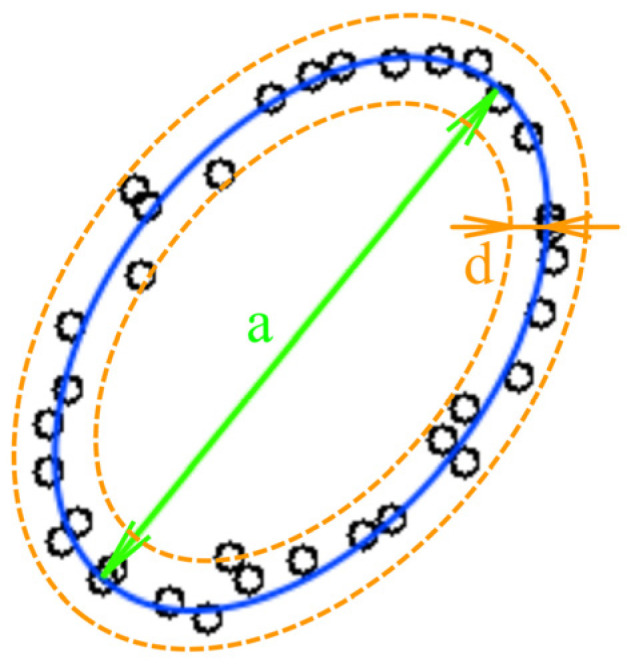
An example of the low-density image.

In our study, we strived to use an easy-to-understand example to investigate the neural network behavior without loss of generality. The first example was chosen for its clarity: the amount of noise (here represented as point location uncertainty) can be estimated visually. Images with low point density (Figure 2) are convenient to distinguish distortions from the object shape.

Figure 2 shows an example of the low-density image. The ideal figure is an ellipse; one can see the deviations of point placements from the perfect ellipse. These deviations are considered as noise or uncertainty. In this paper, we analyze the low-point density image recognition accuracy as a function of the amount of point location uncertainty (further: uncertainty) in the training and test datasets. We also define a way to determine the optimal training parameters.

The research method consists in generating a dataset with pseudo-random low-density point images with different uncertainties and then analyzing the recognition accuracy of these datasets by a trained convolutional neural network, as well as determining the optimal dataset parameters for convolutional neural network training to obtain the best recognition accuracy results.

Robustness estimation of low-density image recognition was investigated in [21]. The robustness study was performed in application to signature verification and resulted in obtaining the miss probability vs. false alarm probability charts with fixed uncertainty. The noise influence on low-density image recognition was also researched in [22]. In both papers, the influence of the training and testing dataset uncertainty on the recognition results was not investigated. The parameter defining the uncertainty measure can be described as ***U*** *= **d/a***, where ***d***—variance and ***a***—linear size of the figure (Figure 2). Further, we consider the training dataset uncertainty as ***U_TR_*** and the testing dataset uncertainty as ***U_TS_***. In other tasks, such as noisy image recognition shown in Figure 1, ***U*** can be described as

***U*** = ***I*_*noise*_/*I*_*info*_**, (1)

where ***I_noise_*** is the average intensity of noise and ***I_info_*** is the average intensity (size) of the meaningful part of the recognized image [17]. In both cases, uncertainty ***U*** describes the ratio of the noisy component of the image to the informative component of the image. The addition of noise of any matter and level can drastically decrease the performance of recognition for a neural network trained on an ideal dataset, so the robustness study should be conducted to avoid this effect by altering the training dataset properties.

The low point density image recognition tasks have already been researched by the authors in a series of papers dedicated to the estimation and prediction of behavior of mobile communication network subscriber groups and complex clusters by analyzing teletraffic and geolocation data [23,24,25]. The locations of subscribers in groups can be considered as sparsely located points on images. Convolutional neural networks have shown their effectiveness in solving this task [26], but the analysis and justification of the applied method’s stability on the initial data uncertainty have not been conducted so far. In [23], the mathematical model describing the subscribers’ cluster shapes was implemented, and it was shown that typical cluster shapes, representing images with low point density, can also be automatically classified by convolutional neural networks.

The exhaustive characteristics of the proposed method stability have not been obtained so far, but there is a good basis to suppose that different uncertainties in datasets can significantly distort the forms, representing subscriber groups, and complicate the recognition of these forms. Thus, the evaluation and optimization of the convolutional neural network robustness in solving the recognition tasks require specific statistical modeling. This task presents a great theoretical interest, since the results of this paper can be applied to all fields of machine learning—the work describes a largely simplified model, thereby summarizing its conclusions for most cases solved by neural networks and other machine learning models.

## 2. Research Plan

**Figure 3:**
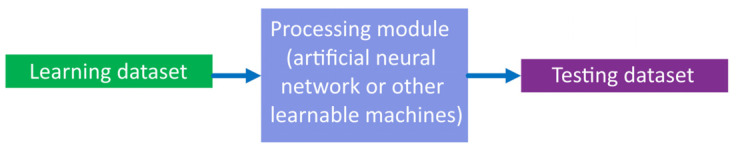
Common structure of supervised learning and inference system.

In a convolutional neural network, the overall supervised learning and inference system structure is as shown in Figure 3.

As stated before, the optimization of the neural network itself, without taking the training dataset influence into account, will generally not provide an exhaustive result and will only allow improving the behavior of the system in some cases. Although the deepening of a neural network (generally, but not applicable in this work because of the simplicity of images, which will be shown further) leads to a better ability of this network to detect and generalize the hidden features [26,27,28], but also creates many problems, such as network resource consumption increase, vanishing gradients problem, etc. Optimization must be performed, taking the properties of all “training dataset-processing module-testing dataset” system components into account.

A widespread traditional approach involves obtaining the fixed test dataset recognition accuracy by a network trained on a fixed training dataset with a given uncertainty ***U*_0_**. This accuracy can be described as a single number, a scalar ***P*_0_**. Accuracy ***P*_0_** is described as follows:

(2) $$P_{0}=\frac{M_{correct}}{M_{total}},$$

where ***M_correct_*** is the number of correctly recognized items in the testing dataset and ***M_total_*** is a total number of items in the testing dataset.

This scalar approach only allows estimating the local properties of the learning-recognition system, but it does not allow estimating the behavior of this system at different data uncertainties. We propose a deeper vector and matrix approach to evaluate the network stability and robustness, which includes the following two sequential steps:

(1) Obtaining an array of test dataset recognition accuracies ***P*** at various test dataset uncertainties ***U_TS_*** with a fixed training dataset uncertainty ***U_TR_***—vector ***P****(**U_TS_**)*.

(2) Obtaining a two-dimensional array of testing dataset recognition accuracies ***P***, depending on their uncertainties and on training dataset uncertainties ***U_TR_*** at the same time—matrix ***P***(***U_TR_***; ***U_TS_***).

Thus, there is an increase in the informativity concerning learning-recognition system robustness and optimality estimation at each following step. With known ***P***(***U_TR_***; ***U_TS_***), we can obtain ***P***(***U_TS_***) and ***P*_0_**:

(3) $$P(U_{TS})=\frac{1}{N_{TR}}\cdot \sum_{U_{TR}}P(U_{TR};U_{TS});$$

(4) $$P_{0}=\frac{1}{N_{TS}}\cdot \sum_{U_{TS}}P(U_{TS}),$$

where ***N_TR_*** is the amount of various learning dataset uncertainties ***U_TR_***, ***N_TS_*** is the amount of various testing dataset uncertainties ***U_TS_***.

**Figure 4:**
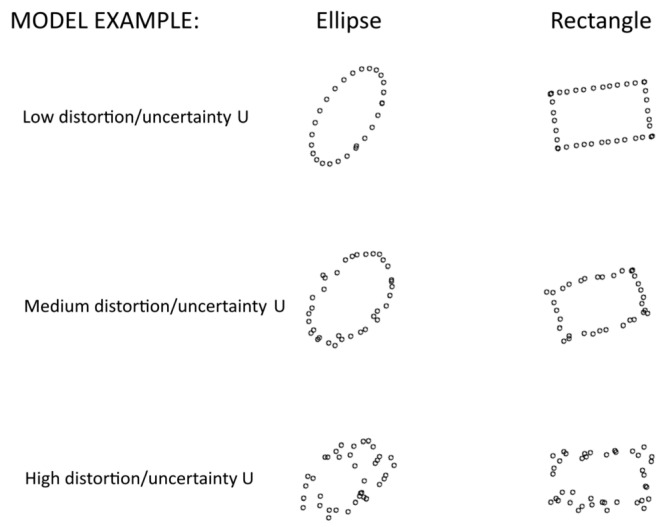
Low-density image model with different point location uncertainties.

A convenient image experiment model was chosen to evaluate the external specifications of the learning and inference system. The mathematical model described in paper [23] allows us to automatically generate the datasets used for convolutional neural network training and testing, and it also allows us to set various uncertainty parameters (for example, the point position offset relative to the shape vector model (Figure 4). This fact allows evaluating the convolutional neural network stability to the changing dataset uncertainty parameters and to evaluate the neural network characteristics in the conditions of the factors, increasing the input data distortions. This fact allows evaluating the convolutional neural network stability to the changing dataset uncertainty parameters and evaluating the neural network characteristics in the conditions of the presence of the factors, increasing the input data distortions.

**Figure 5:**
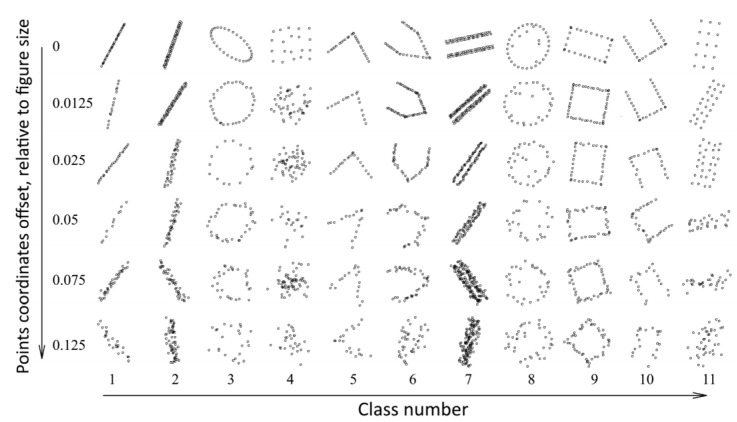
Examples of generated images with various distortions (uncertainties).

To evaluate the trained neural network noise immunity characteristics in paper [23], we generated 200 datasets with different uncertainties (Figure 5).

As one can see in Figure 5, the uncertainty of the coordinates of individual points distorts the image, but the common shapes retain their characteristic features.

## 3. Image Generation and Distortion Model

**Figure 6:**
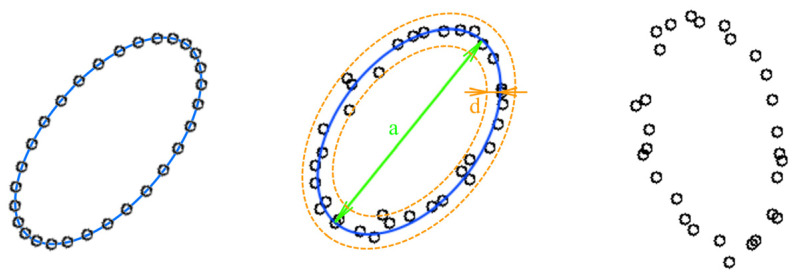
Image generation.

Figure 6 shows our way of offset implementation—as the uncertainty increases, the images are distorted more strongly. Figure 4 shows the result of image generation. The result of the modeling is a set of images with a resolution of 256 × 256 pixels. This image generation model is based on its interpretation ease; research results in the future can be generalized toward a wider class of tasks. The chosen resolution is sufficient to provide necessary accuracy in the representation of distorted images without affecting the convolutional neural network speed and complexity and the array size. The resulting image generation and distortion model can be described as follows (Figure 6):

1. Creating a shape vector model with uniformly distributed points (the number of points is random and distributed uniformly in some range).
2. Adding (to every point) the individual position offset described with a Gaussian random distribution (the variance of the distribution sets the amount of uncertainty). The Gaussian random distribution is well-suited to describe the uncertainty that occurs due to a set of different reasons. The uncertainty is measured relative to the shape vector model size. For example, an uncertainty value of 0.1 relative units says that the position of points may vary within 0.1 of the maximum linear size of the figure.
3. The resulting figure is rotated at a random angle.

## 4. Structure of Neural Network

**Figure 7:**
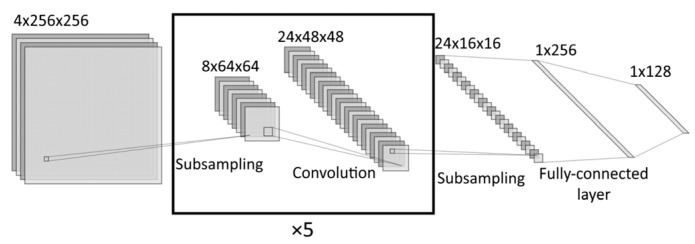
Structure of CNN.

This architecture is one of the simplest and popular [29]. The convolutional network consists of alternating convolutional and subsampling layers (Figure 7), and the training process consists of repeatedly presenting a training dataset to the network (each iteration is called an epoch) and correcting the synaptic weights of the network at each iteration. When the synaptic weights stabilize, the mean error on the whole training set is minimized, the network can be considered trained. Images obtained from the image generation module merged to create the training dataset.

## 5. Estimation of the Recognition Quality Dependence on the Amount of Uncertainty

First, we used a neural network to recognize each testing dataset with its amount of uncertainty ***U_TS_***, which allowed us to determine the dependence of the recognition accuracy on uncertainty in the testing dataset ***P***(***U_TS_***).

To obtain more information, we trained two independent CNNs with identical structures on two training datasets, with two different amounts of uncertainty: ***U_TR_*** = 0 and ***U_TR_*** = [0…0.025]. The hyperparameters remained unchanged. Separate datasets with image sequence randomizations were created for each experiment. The rule for dataset generation is described in [23]. Initial weights of CNNs were randomized as run-to-run. Trained CNNs were used for the recognition of separately generated datasets containing images with various uncertainties ***U_TS_***. All recognition probabilities ***P*** obtained in these series of simulations were averaged over all series of experiments with fixed ***U_TS_***.

**Figure 8:**
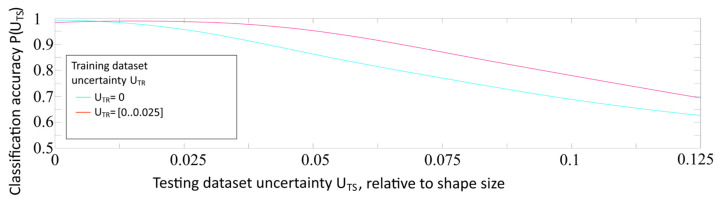
Dependence of the recognition accuracy on the amount of testing dataset uncertainty ***U_TS_***, obtained by a network trained with uncertainty ***U_TR_*** = 0 and ***U_TR_*** = (0..0.025).

As a result, two arrays of recognition accuracies, as functions of testing dataset uncertainty ***P***(***U_TS_***), were obtained. The results of this experiment are summarized in the graph shown in Figure 8.

The comparison of two graphs allowed us to draw three main conclusions:

1. At ***U_TR_*** = 0 the accuracy curve is monotonous. It confirms the consistency and robustness of the chosen model.
2. At ***U_TR_*** = (0…0.025) the accuracy curve is “no more” monotonous; it shows a small drop of accuracy below ***U_TS_*** = 0.01. This phenomenon shows that changing the uncertainty proportions in the training dataset may affect the recognition of ideal images.
3. The maximum accuracy is achieved at ***U_TR_*** = 0 and ***U_TS_*** = 0, but the integral (overall) accuracy at all considered values of ***U_TS_*** is achieved at ***U_TR_*** = (0…0.025). This phenomenon can be explained by the limited ability of the neural network, trained only on perfect images without uncertainty, to generalize the features presented in the distorted examples with uncertainty.

**Figure 9:**
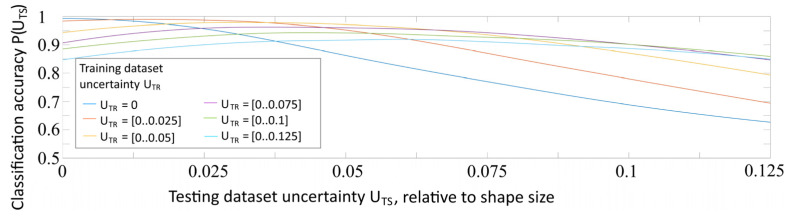
Dependency of recognition accuracy on the amount of uncertainty, obtained by the networks trained on datasets with ***U_TR_*** varying from 0 to 0.125 stepping 0.025.

It is well known that the characteristics of the training dataset strongly affect the neural network training quality and the accuracies of their future new dataset recognition tasks [30]. To optimize the convolutional neural network training (to discover the optimal training dataset parameters to improve the recognition quality of images with different uncertainties) in this paper, we conducted experiments by training the convolutional neural network using datasets with various amounts of uncertainty. From the graphs shown in Figure 7 and Figure 8, we cannot make unequivocal conclusions about training optimality. In our research, we conducted more training experiments, thereby “unfolding” the results in a new dimension (amount of uncertainty in the training dataset). Figure 9 shows the dependencies of recognition accuracy on the amount of uncertainty ***U_TS_***, obtained by the networks trained on datasets with ***U_TR_***, varying from 0 to 0.125 in increments of 0.025, shown in one graph for clarity.

Figure 9 shows that the maximum recognition accuracy is achieved at ***U_TS_*** ≤ ***U_TR_***. Moreover, an analysis of the graphs in Figure 9 shows that for ***U_TR_*** ≥ 0.025, the accuracy graphs change their shapes from monotonic to non-monotonic, indicating that the training is not optimal and is non-robust. The dependence of ***P****(**U_TS_**)* should be monotonous, $dP/dU_{TS}\leq 0$ due to the fall of the meaningful information quantity with rising uncertainty. This rule may be used as the criterion for training correctness and robustness.

## 6. Evaluating the Dependence of Recognition Quality on Uncertainties in Testing (*U_TS_*) and Training (*U_TR_*) Datasets

**Figure 10:**
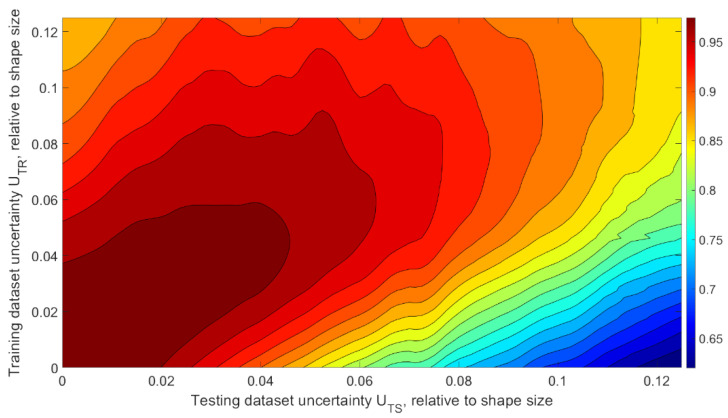
Dependency of recognition accuracy on the amount of training and testing dataset uncertainties ***U_TR_*** and ***U_TS_***.

The results obtained at this stage (presented in the graphs in Figure 9) prompted us to conduct comprehensive research on the network behaviors under changing training dataset uncertainty ***U_TR_***. For a more detailed analysis of the image recognition accuracy, we generated a manifold of training datasets with different uncertainties $U_{TR\;i}=d_{i}/a$. Individual copies of the convolutional neural network shown in Figure 7 were trained, and their weights were obtained for each training dataset. Then, each trained network was made to recognize each test dataset with different uncertainties $U_{TS\;j}=d_{j}/a$. It allowed us to obtain a two-dimensional array (matrix) of recognition accuracy, depending on the training and testing dataset uncertainties $P=P\left(U_{TR};U_{TS}\right)$. The resulting recognition accuracy matrix contains complete information about the external training-recognition system specifications and can be used to assess the system consistency and robustness (Figure 10).

As one can see in Figure 10, the network better recognizes data with the same or a lower uncertainty, which was used in the training dataset, which confirms the correctness of the training because the network better recognizes data with similar statistical characteristics than those used during training. The recognition accuracy achieved by the network trained by the dataset with high ***U_TR_*** drops on data with low ***U_TS_***; it is caused by changing proportions of meaningful and noisy data proportions during training. The “ripple” that can be seen in the graph is an effect of the limited dataset and should be considered a statistical inaccuracy. Since each recognition system in real life has its minimum accuracy requirements [31], it is necessary to analyze the recognition accuracy for different values of the minimum recognition accuracy thresholds.

## 7. Cumulative Recognition Accuracy at Different Thresholds

**Figure 11:**
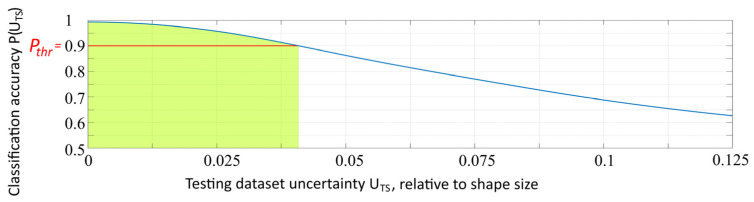
The area that includes the values of testing dataset uncertainty with recognition accuracy higher than 90%.

In practical tasks solved by neural networks, it is often not necessary to recognize data with extremely high distortions. Moreover, there are often minimal classification/recognition accuracy requirements to the developed solutions that use neural networks. Often, in practice, there is a necessity to gain a high enough “certainty” of the system. To obtain more valuable results, we selected areas that included the values of the testing dataset uncertainty with a recognition accuracy higher than ***P_thr_,*** in which the recognition accuracy was higher than the selected thresholds, which allowed estimating the acceptable coordinate uncertainties to provide the necessary recognition accuracy. Figure 11 shows the area that included the values of the testing dataset uncertainty ***U_TS_*** that provided recognition accuracy ***P_thr_*** higher than 90%.

The highlighted area in Figure 11 is calculated as

(5) $$Q(P_{thr})=\sum_{U_{TS}=U_{TS}^{\min},P\geq P_{thr}}^{U_{TS}=U_{TS}^{\max},P\geq P_{thr}}P(U_{TS}).$$

The task of determining the optimal training dataset parameters to obtain the required recognition accuracy above threshold ***P_thr_*** arises. For each network trained on datasets with different uncertainties, we obtained the integral recognition accuracy values ***Q*** at different thresholds:

(6) $$Q(U_{TR};P_{thr})=\sum_{U_{TS}=U_{TS}^{\min},P\geq P_{thr}}^{U_{TS}=U_{TS}^{\max},P\geq P_{thr}}P(U_{TR};U_{TS}).$$

**Figure 12:**
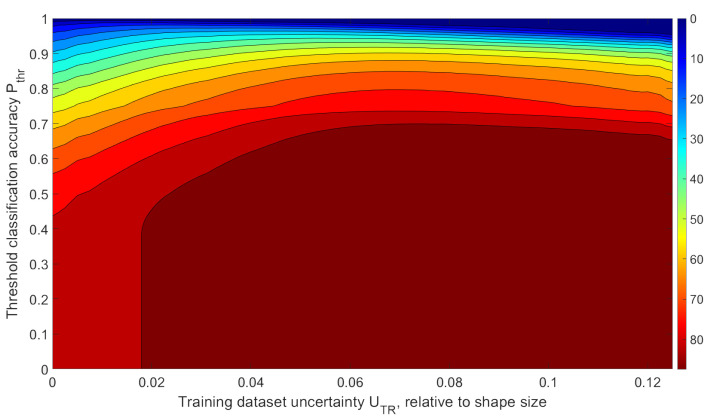
Dependence of the integral value of the recognition accuracy ***Q*** for all $P>P_{thr}$ on $P_{thr}$ and training dataset uncertainty ***U_TR_***.

In this case, ***Q*** is an integral value of the classification accuracy for all test datasets (with all uncertainties), for which the recognition accuracy exceeded threshold ***P*** *≥* ***P_thr_***. The obtained data are summarized in the graph shown in Figure 12 below.

This graph represents the integral (overall) average rate of correct recognition for data giving the recognition probability greater than ***P_thr_***, depending on ***P_thr_*** and training dataset uncertainty ***U_TR_***. New data recognized with accuracies lower than the required ***P_thr_*** were not counted. The colors in the graph show areas with identical mean recognition accuracies for all $P>P_{thr}$**.** There is always an optimal training dataset uncertainty ***U_TR_***, depending on the lower threshold of the required recognition accuracy.

**Figure 13:**
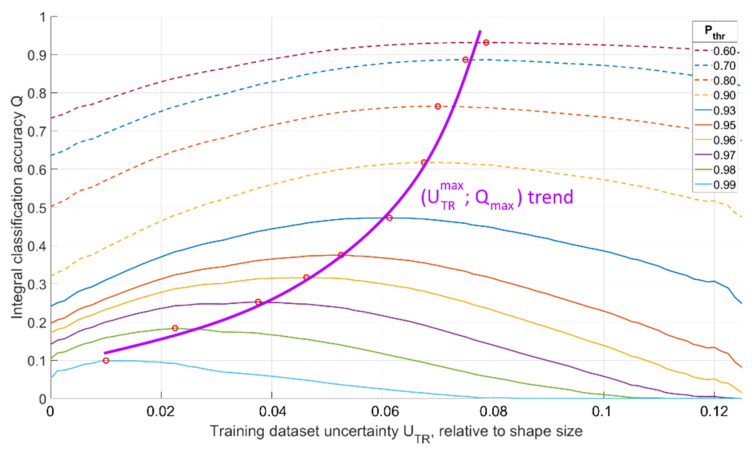
Dependence of the integral recognition accuracy ***Q*** on training dataset uncertainty ***U_TR_*** for various ***P_thr_*** and optimal values of training dataset uncertainty ***U_TR_*** for different recognition accuracy thresholds ***P_thr_***.

Using Figure 12, one can determine the optimal training dataset uncertainty ***U_TR_*** needed to achieve maximal integral recognition accuracy ***Q*** for all data with local recognition probability exceeding the threshold ***P*** ≥ ***P_thr_***. It can be illustrated in Figure 13.

Assuming that the optimal training dataset at the required threshold of the minimum classification accuracy is the dataset that gives the highest value of integral classification accuracy ***Q***, the graph in Figure 13 is convenient for determining the optimal training dataset uncertainty ***U_TR_***. If we analyze the dependence of the integral recognition accuracy ***Q*** on the training dataset uncertainty ***U_TR_*** at a fixed threshold ***P_thr_***, we obtain a graph with a clear maximum, the position of which will indicate the optimal value of training dataset uncertainty ***U_TR_*** (Figure 13, ***Q_max_*** for various ***P_thr_*** are shown with red dots). An analysis of Figure 13 allows us to conclude that training the network with optimal ***U_TR_*** for fixed ***P_thr_*** significantly increases the integral recognition accuracy compared to training the network with an ideal dataset with ***U_TR_*** = 0. For example, for ***P_thr_*** = 0.9, ***Q_max_*** exceeds ***Q*_0_** by 94% (***Q_max_*** = 0.62 is obtained at ***U_TR_*** = 0.068 and ***Q*_0_** = 0.32 is obtained at ***U_TR_*** = 0).

## 8. Noisy Images Recognition

**Figure 14:**
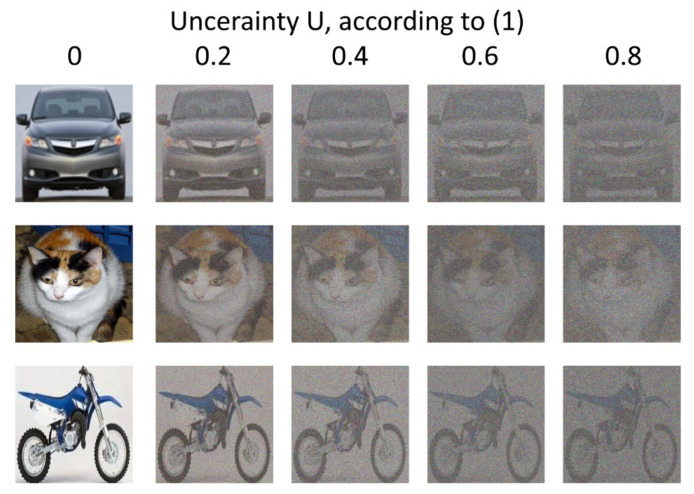
Examples of images with various amounts of noise added.

To generalize the results of this study, we conducted a simulation with different types of images and noise using the same CNN structure and the same approach involving an analysis of $P\left(U_{TR};U_{TS}\right)$ dependence. The examples of images are shown in Figure 14. We added white Gaussian noise with mean **μ** = 0 and various standard deviations **σ** to the images to generate separate datasets for training and recognition. The uncertainty ***U*** is therefore defined via (1), where ***I_noise_*** = **σ**.

We used a convolutional neural network identical to the one described before to solve the noisy image classification task. Five datasets for training were generated. The training dataset parameters are described below:

(1) The first dataset had ***U_TR_*** = 0 for all images (no noise was added).
(2) The second dataset was divided into three parts containing equal numbers of images; the first part had ***U_TR_*** = 0, the second part had ***U_TR_*** = 0.04, the third part had ***U_TR_*** = 0.08.
(3) The third dataset was divided into three parts containing equal numbers of images; the first part had ***U_TR_*** = 0, the second part had ***U_TR_*** = 0.12, the third part had ***U_TR_*** = 0.16.
(4) The fourth dataset was divided into three parts containing equal numbers of images; the first part had ***U_TR_*** = 0, the second part had ***U_TR_*** = 0.2, the third part had ***U_TR_*** = 0.4.
(5) The fifth dataset was divided into three parts containing equal numbers of images; the first part had ***U_TR_*** = 0, the second part had ***U_TR_*** = 0.4, the third part had ***U_TR_*** = 0.8.

Thus, four of five datasets had various amounts of noise added to the original images.

Five independent CNNs with identical structures were trained using five datasets described above. The hyperparameters remained unchanged. Separate datasets with image sequence randomizations were created for each experiment. Initial weights of CNNs were randomized run-to-run. Trained CNNs were used for recognition of separately generated testing datasets containing images with various uncertainty values ***U_TS_***. Testing datasets had homogenous structures: all images in each dataset had the same amount of additional noise, giving us fixed ***U_TS_*** for the whole dataset. All recognition probabilities ***P*** obtained in these series of simulations were averaged over all series of experiments with fixed ***U_TS_***.

**Figure 15:**
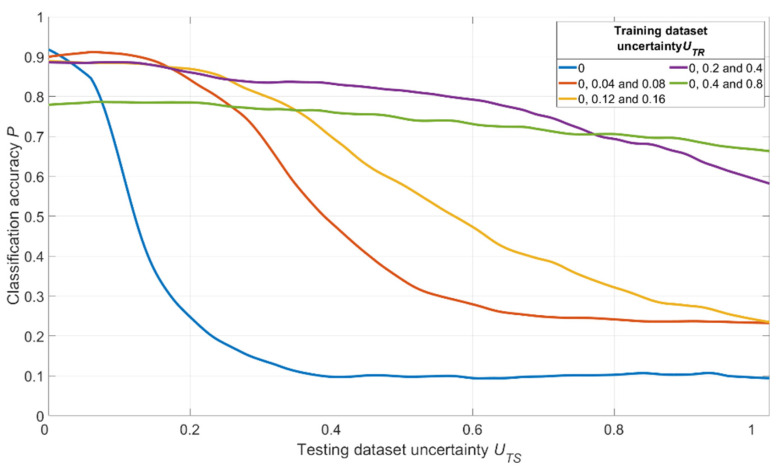
Dependence of the recognition accuracy on the amount of testing dataset uncertainty ***U_TS_***, obtained by five CNNs trained with various uncertainties ***U_TR_*** shown in the image.

The simulation results are shown in Figure 15.

**Figure 16:**
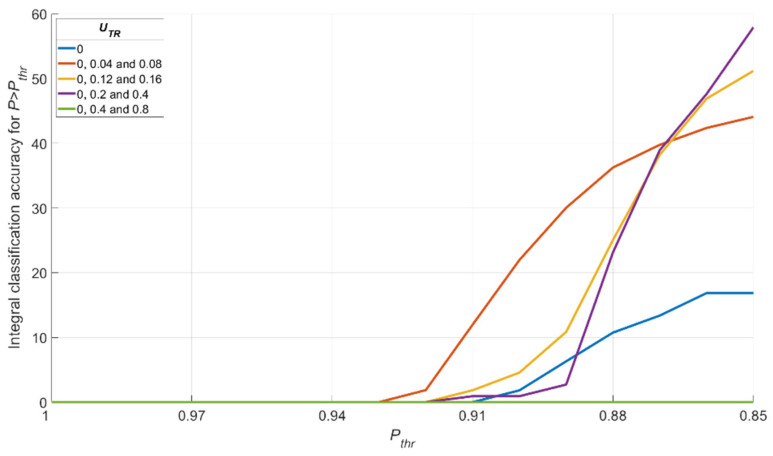
Dependence of the integral value of the recognition accuracy ***Q*** for all $P>P_{thr}$ on $P_{thr}$ for various training dataset uncertainties ***U_TR_***.

The results shown in Figure 15 and Figure 16 allow us to state that moderate training dataset uncertainty ***U_TR_*** should be optimal for recognition of noisy images with high threshold recognition probability ***P_thr_***. For the current example, the optimal training dataset is the second one, having ***U_TR_*** = {0; 0.04; 0.08} for ***P_thr_*** > 0.87. A further increase of ***U_TR_*** leads to the fall of integral classification accuracy for high ***P_thr_***. This result allows us to generalize the confirmation of existence of optimal training dataset uncertainty for these types of data.

## 9. Additional Types of Distortion

**Figure 17:**
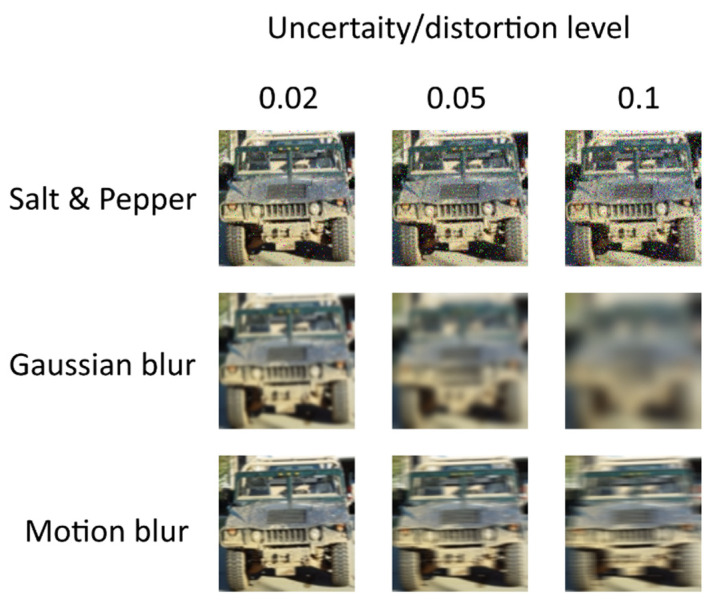
Examples of images with various amounts of salt and pepper noise, Gaussian blur and motion blur added.

To check the correctness of our findings, we conducted a series of experiments with various types of image distortions. We conducted these series of simulations with different types of images and noise/distortions using the same CNN structure and the same approach, involving analysis of $P\left(U_{TR};U_{TS}\right)$ dependence. The examples of images are shown in Figure 17.

**Figure 18:**
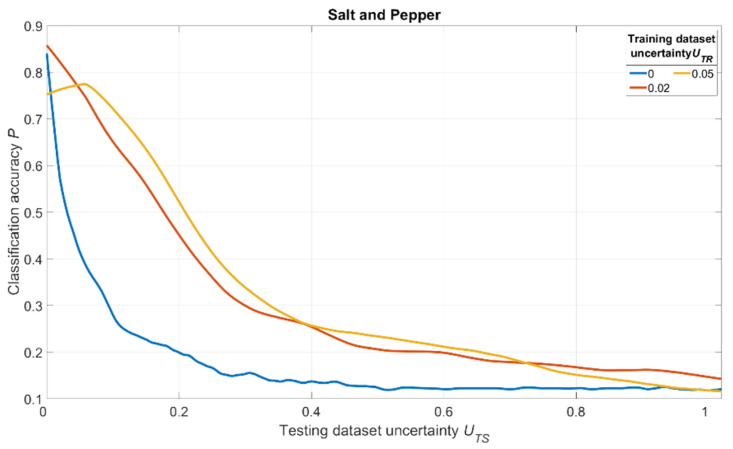
Dependence of the recognition accuracy on the amount of testing dataset uncertainty ***U_TS_***, obtained by three CNNs trained with various uncertainties ***U_TR_*** shown in the image. The uncertainty is produced by adding salt and pepper noise and calculated using (1).

**Figure 19:**
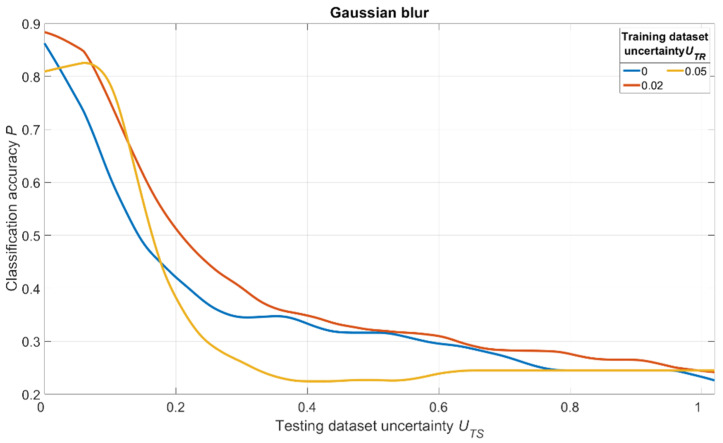
Dependence of the recognition accuracy on the amount of testing dataset uncertainty ***U_TS_***, obtained by three CNNs trained with various uncertainties ***U_TR_*** shown in the image. The uncertainty is produced by adding Gaussian blur and calculated using (7).

**Figure 20:**
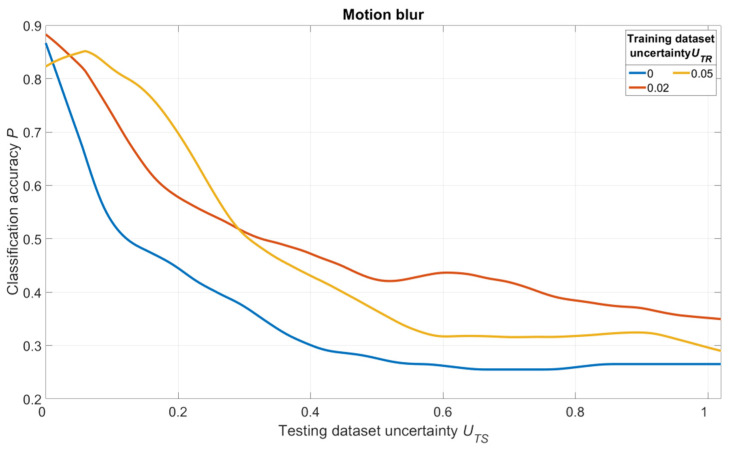
Dependence of the recognition accuracy on the amount of testing dataset uncertainty ***U_TS_***, obtained by three CNNs trained with various uncertainties ***U_TR_*** shown in the image. The uncertainty is produced by adding motion blur and calculated using (7).

The simulation results are shown in Figure 18, Figure 19 and Figure 20. For salt and pepper noise, the uncertainty was calculated using Formula (1), where ***I_noise_*** is a number of “noise” pixels and ***I_info_*** is the total number of pixels in the image. For Gaussian and motion blur, uncertainty was calculated as follows:

(7) $$U=S_{kernel}/S_{image},$$

where ***S_kernel_*** is the size of Gaussian and motion filter kernels and ***S_image_*** is the size of the image.

The analysis of Figure 18, Figure 19 and Figure 20 shows one tendency: independently on noise/distortion type during training; its amount influences the recognition accuracy in the same way. Using images without additional uncertainty for training (***U_TR_*** = 0, blue curves) leads to a fast decrease of recognition accuracy with the rise of ***U_TS_***. This fact tells us that a CNN trained in such a manner would be vulnerable to adversarial attacks. The results also show that a moderate training dataset uncertainty ***U_TR_*** (red curves) in case of salt and pepper noise, as well as of Gaussian and motion blur, should be optimal for recognition of noisy/distorted images without loss of accuracy for original image recognition. This result allows us to generalize the confirmation of the existence of optimal training dataset uncertainty for these types of data, noise, and distortion.

Thus, we conducted simulations under a variety of conditions, and all of them show the same regularity: there exists an optimal amount of uncertainty of various physical natures that, when applied to the training dataset, lead to significant improvements of trained CNN noise immunity and overall recognition quality. The excessive amount of training dataset uncertainty (yellow curves in Figure 18, Figure 19 and Figure 20) makes the training non-robust. It can be seen as a non-monotonous character of the corresponding (yellow) noise immunity curves (growth of recognition accuracy with increasing ***U_TS_***).

## 10. Conclusions

This study shows that the amount of uncertainty in the training dataset ***U_TR_*** significantly affects the recognition accuracy itself and the dependence of the recognition accuracy on the uncertainty in the testing dataset ***U_TS_***. We analyzed the recognition accuracies of multiple datasets with different uncertainties and obtained the dependence of recognition accuracy on the training dataset uncertainty. The existence of an optimal (in terms of recognition accuracy) amount of uncertainty in the training dataset (for the neural networks working with undefined uncertainty data) was hypothesized and proven for various types of images and noise. We have shown that the determination of this optimum can be performed using statistical modeling. Training the network using the dataset with optimal uncertainty ***U_TR_*** provides a significant increase of recognition accuracy compared to training on the ideal dataset. The CNN learns not to use the noise/distortion as features during training because noise/distortion does not help the CNN distinguish different images. Therefore, it enhances the generalization ability of the CNN and its immunity to adversarial attacks. At the same time, excessive noise/distortion ruins the training, leading to recognition accuracy decrease. This finding can be used to improve recognition quality just by adding some (optimal) amounts of noise to the training dataset.

The obtained results are applicable to convolutional neural networks with common structures and different types of data uncertainty (Gaussian noise, distortion of point locations, salt and pepper noise, Gaussian and motion blur, etc.). Future work will be devoted toward expanding the results on different neural network structures and different tasks (for example, object detection). We hope to find an analytical solution for optimal training dataset uncertainty determination without massive statistical simulations.

## Data Availability

Not applicable.
